# Clinical evaluation of digital versus conventional impression in edentulous patients with flabby ridges: a randomized controlled clinical trial

**DOI:** 10.1186/s12903-025-07524-8

**Published:** 2026-01-12

**Authors:** Dina Mohamed Elawady, Ahmed Yehia Abdel-Moneim, Wafaa Ibrahim Ibrahim, Sherin Matta

**Affiliations:** 1 Department of Prosthodontics, Faculty of Dentistry, October University for Modern Sciences and Arts, 6th of October City, Egypt; 2 Department of Prosthodontics, Faculty of Oral and Dental Medicine, Delta University for Science and Technology, International Coastal Rd, Dkahleya, Egypt

**Keywords:** Maxillary completely edentulous, Flabby ridge, Intraoral scanning, Window impression, Oral health related quality of life, Retention

## Abstract

**Background:**

The optimal impression technique for flabby ridge cases remains ambiguous despite the advancements in impression techniques. This randomized controlled trial (RCT) aimed to compare the Oral Health Related Quality of Life (OHRQoL) and retention of maxillary dentures fabricated from intraoral scanning (IOS) versus window impression technique in completely edentulous patients with maxillary flabby ridges.

**Methods:**

The study utilized a crossover design, where participants were randomly assigned to receive conventional complete dentures fabricated by using two different techniques for complete denture fabrication: one utilizing conventional impression method (Group I dentures) and the other employing digital impression technique (Group II dentures). OHRQoL and retention of maxillary dentures were evaluated at baseline (T0), 3 months (T3), and after 6 months (T6). A new complete denture was delivered for each participant after 6 months, with those initially receiving Group I dentures transitioning to Group II dentures, and vice versa. Comparison between groups using paired t-test, while comparison between different time points was performed by using Repeated Measures ANOVA test followed by Tukey’s Post Hoc test. The significant level was set to be at *P* ≤ 0.05.

**Results:**

Both groups reported improvement in OHRQoL with no significant difference between groups at six months follow-up period (*P* < 0.0001). Additionally, both groups reported a decline in denture retention at the follow up periods with no significant difference between groups at six months follow-up period (*P* < 0.0001).

**Conclusion:**

Digital impression is a viable alternative to conventional impression in the fabrication of complete dentures for edentulous arches with flabby ridges with similar outcomes in terms of OHRQoL and denture retention.

**Trial registration:**

The study protocol was retrospectively registered and posted on the ClinicalTrials.gov public website (ClinicalTrials.gov identifier: NCT07108322) on August 7, 2025.

**Supplementary Information:**

The online version contains supplementary material available at 10.1186/s12903-025-07524-8.

## Background

Edentulism is a major global public health issue that mainly affects the elderly [1]. A person’s speech, facial appearance, nutrition, masticatory efficiency, and overall health are all significantly impacted by edentulism [2]. In order to restore these lost functions and enhance patient comfort and appearance, complete dentures are usually the practical and economical prosthetic option of choice [3]. 

However, the ongoing physiological process of residual ridge resorption frequently affects the ability of complete dentures to perform successfully. Flabby ridge is a clinical condition that makes complete denture fabrication and retention more difficult [4]. Usually found in the maxillary region, a flabby ridge also referred to as a hypermobile or displaceable ridge is characterized by an excess of mobile soft tissue that replaces the alveolar bone, most commonly found in the anterior region of the maxilla [5]. During the impression-making, the mobile tissue is easily deformed which may compromise denture stability, support, and retention. This leads to poor function of the prosthesis and discomfort for the patient. Achieving optimum retention is an important factor in the success and patient acceptance of complete dentures [6]. 

Traditional impression techniques have been the most used method for making complete dentures [7]. In order to minimize tissue displacement; these techniques usually require a number of steps, materials, and specific adjustments when recording flabby ridges. Although a window impression technique aims to record flabby tissues without displacement, it has some clinical challenges. For example the difficulty of applying and controlling the low-viscosity impression materials uniformly, is a significant limitation. Gravity, especially in the maxillary arch, can exacerbate the problem [8]. If this lack of control causes the flabby tissues to be displaced or distorted; the accuracy of the impression may be at risk [9]. 

IOS recently became an acceptable digital alternative for recording edentulous arches during construction of complete denture [10]. Some of the benefits of IOS are reduced chair time, less gag reflex, removal of impression materials, which may improve patient comfort, and facilitate communication with dental laboratories [11, 12]. 

A direct comparison between IOS and traditional impression techniques is needed due to a growing number of flabby ridges in edentulous patients. [12–15] This randomized controlled clinical trial aimed to evaluate and compare the clinical outcomes, specifically OHRQoL and retention of maxillary dentures fabricated using IOS versus window impression techniques in completely edentulous patients with maxillary flabby ridges.

The null hypothesis of this study states that there is no significant difference in OHRQoL and retention of maxillary dentures fabricated from IOS and those fabricated using window impression technique in edentulous patients with maxillary flabby ridges.

## Materials and methods

### Ethical approval

All participants provided informed consent upon inclusion in the trial, which was approved by the Research Ethics Committee of the Faculty of Oral and Dental Medicine, Delta University for Science and Technology (approval no. FODMRC-2023.00111) and was conducted in accordance with the Helsinki Declaration of 1975, as revised in 2013. 

### Registration

**Fig. 1:**
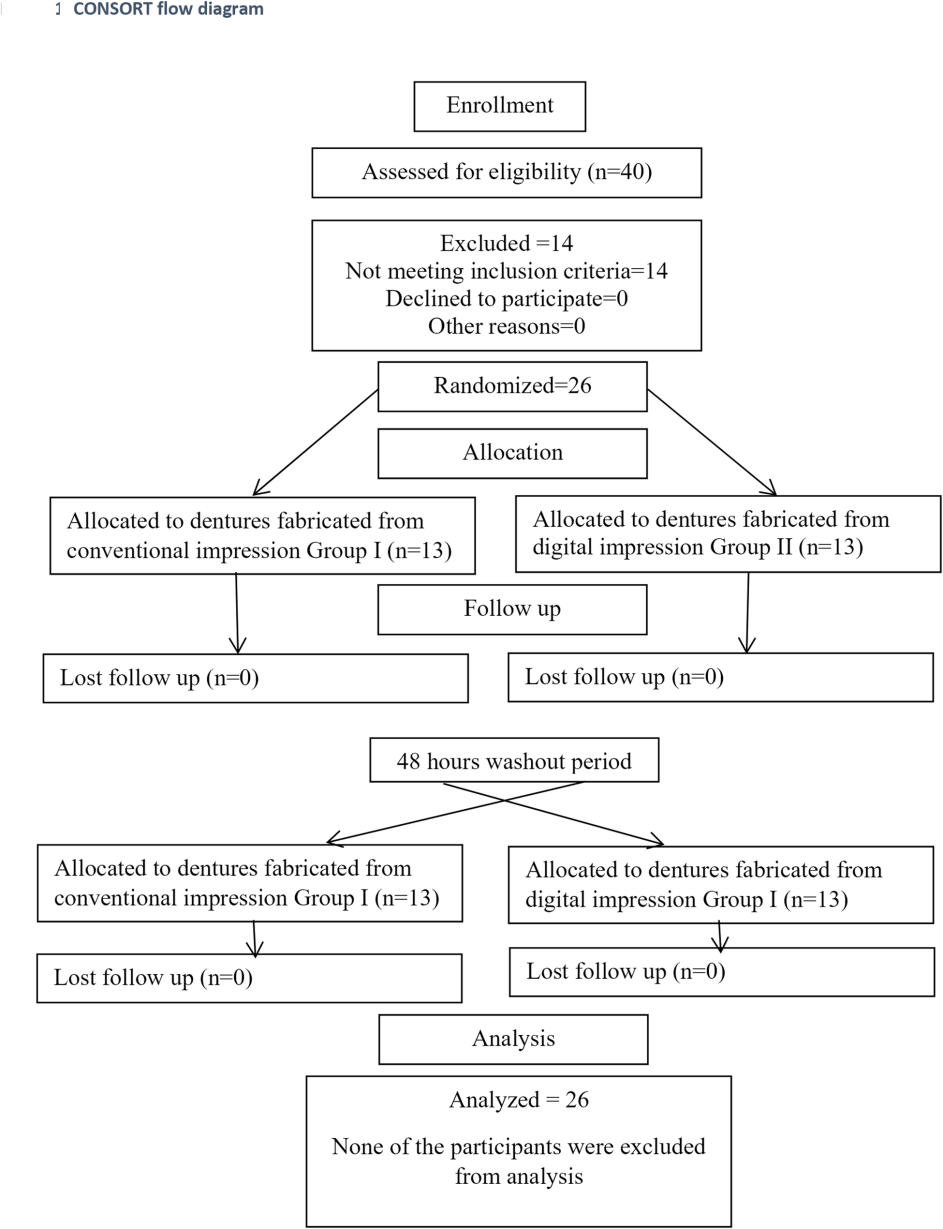
CONSORT flow chart

The study protocol was registered and posted on the ClinicalTrials.gov public website (ClinicalTrials.gov identifier: NCT07108322). Enrollment of participants began on March 30, 2023 and the study was completed on June 25, 2025 Participants enrolled in the study are shown in the Consolidated Standards of Reporting Trials (CONSORT) statement of the flow chart (Fig. 1).

### Sample size calculation

The sample size was calculated using PS: Power and Sample Size Calculation software (version 3.1.2; William D. Dupont and Walton D., Vanderbilt University, Nashville, TN, USA). Based on a previous study (Rostom & ElKhashab, 2024) [13], where the mean ± standard deviation of patient satisfaction VAS scores was 75 ± 15 for conventional impressions, a 12-point improvement (Δ) with digital techniques was estimated. For this crossover design, accounting for within-subject correlation (ρ = 0.65), a minimally required sample size of 22 patients was determined to achieve 90% power at α = 0.05 using a paired t-test. This was increased to 26 patients (13 per sequence group) to accommodate potential dropouts.

### Participants and setting

**Fig. 2:**
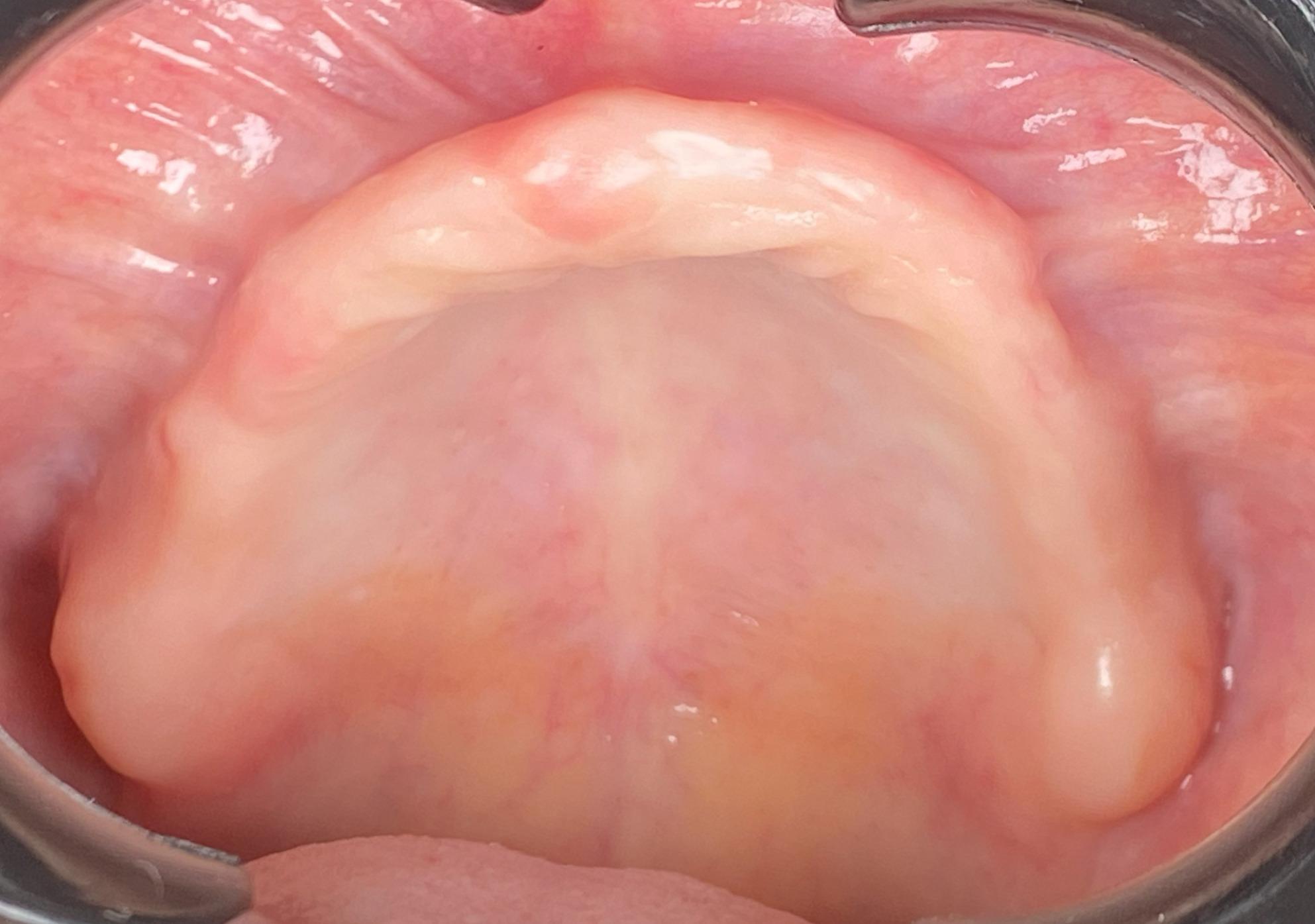
Flabby ridge intra oral photo

A total of 26 participants who met all pre-defined inclusion criteria were consecutively enrolled in the study. All eligible patients presenting to the outpatient clinic at the Department of Prosthodontics, Delta University for Science and Technology, during the defined recruitment period were screened for inclusion. After understanding the aim of the study and providing informed consent, patients were included according to the following criteria; they had to be having a completely edentulous maxillary and mandibular arches; with a maxillary flabby ridge localized to the premaxillary region (Fig. 2), with age ranging between 50 and 75 years, in systemic good health (ASA-1/ASA-2), free from infectious diseases, and being willing to cooperate throughout the data collection process. Conversely, individuals were excluded from the study if they were partially edentulous, failed to understand the study’s procedures and objectives, exhibited ridge irregularities or scarring, or had limited mouth opening.

### Design

The study utilized a crossover design, where participants were randomly assigned to receive complete dentures fabricated by using two different techniques for complete denture fabrication: Group I dentures were fabricated using the conventional window technique impression and Group II denture were fabricated using Digital impression technique [14]. A new complete denture was delivered for each participant after 6 months, with those initially receiving Group I dentures transitioning to Group II dentures, and vice versa. 

### Randomization

The randomization sequence was generated by a statistician using a computer-generated list of random numbers at a 1:1 allocation ratio, independent of the study’s clinical team. A non-clinical assistant, who was not involved in patient recruitment or assessment, prepared the opaque, sequentially numbered, sealed envelopes and was responsible for their safekeeping in a locked cabinet. The Principal Investigator (D.E.) was responsible for screening and enrolling eligible participants. Assignment was performed by an independent dental assistant immediately prior to implementing the assigned impression technique by opening the next sequential envelope, thus ensuring adequate allocation concealment.

### Blinding

Patient blinding was maintained because the dentures for both the test and control groups were made of the same acrylic material and were polished to be clinically indistinguishable in appearance, weight, and fit to avoid any obvious physical cues. The outcome assessors (for OHIP-EDENT-19 and retention) were kept rigorously unaware of the participant’s assignment sequence. They had no access to the randomization list and did not participate in the fabrication, insertion, or post-insertion adjustment of the dentures. They performed all assessments based solely on the patient’s ID and were not informed of which denture the patient was currently wearing at the time of assessment.

### Interventions

For Group I dentures, Window Technique Impression was employed to make the final impression, and the sequence of steps was as follows: The primary alginate impression (Cavex CA37; Cavex Holland BV) of the edentulous maxilla was made with stock edentulous trays. Anterior maxillary flabby tissue was palpated and identified intraorally using large ball burnisher and outlined with an indelible pencil. The impression was repositioned in the patient’s mouth and the markings made with an indelible pencil were transferred onto the impression surface. The impression was then poured using Type III dental stone (Kulzer Type III dental stone, Kulzer GmbH), and one sheet of modeling wax (Cavex; Cavex Holland BV) was adapted as a spacer.

**Fig. 3:**
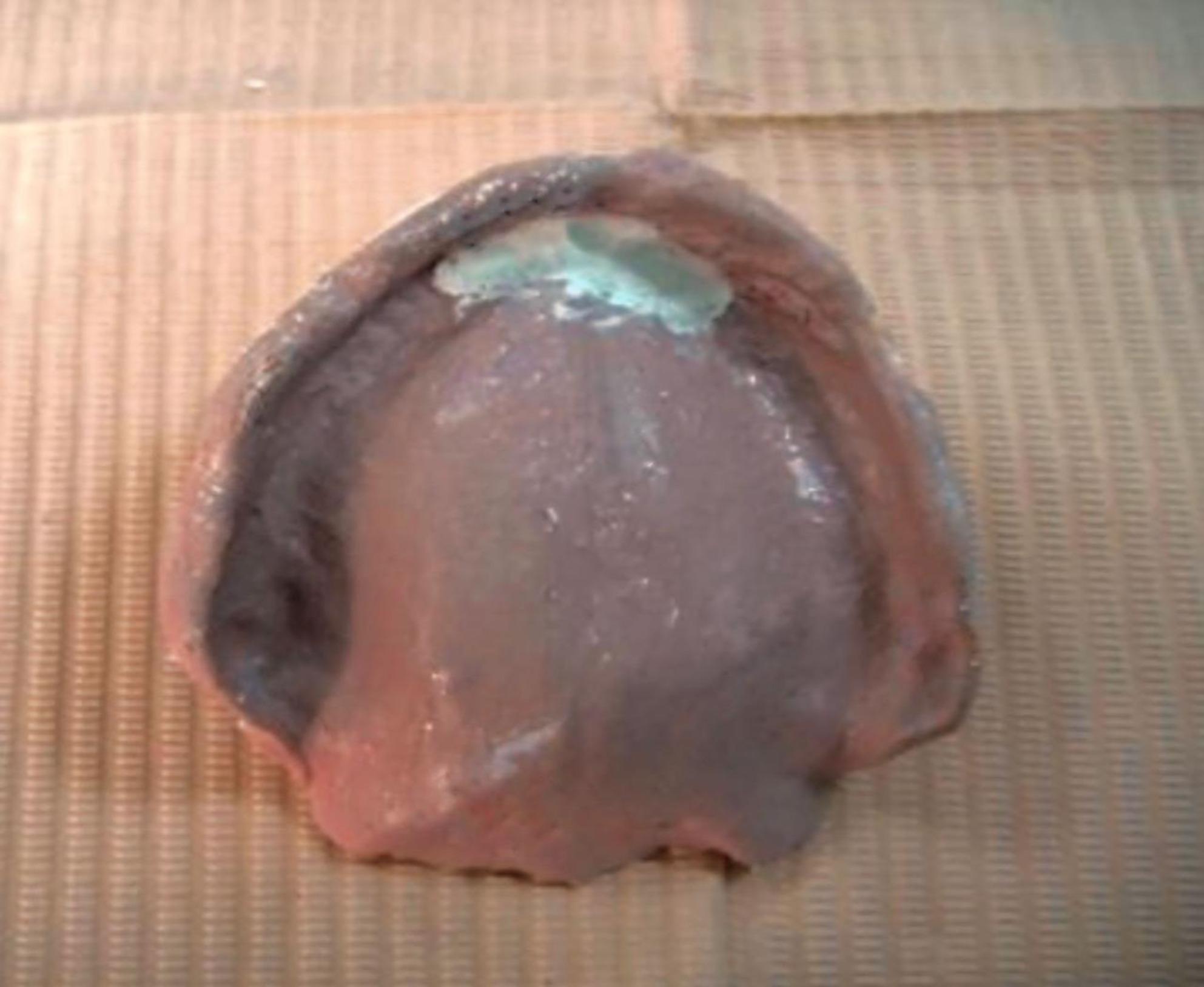
Window technique impression

**Fig. 4:**
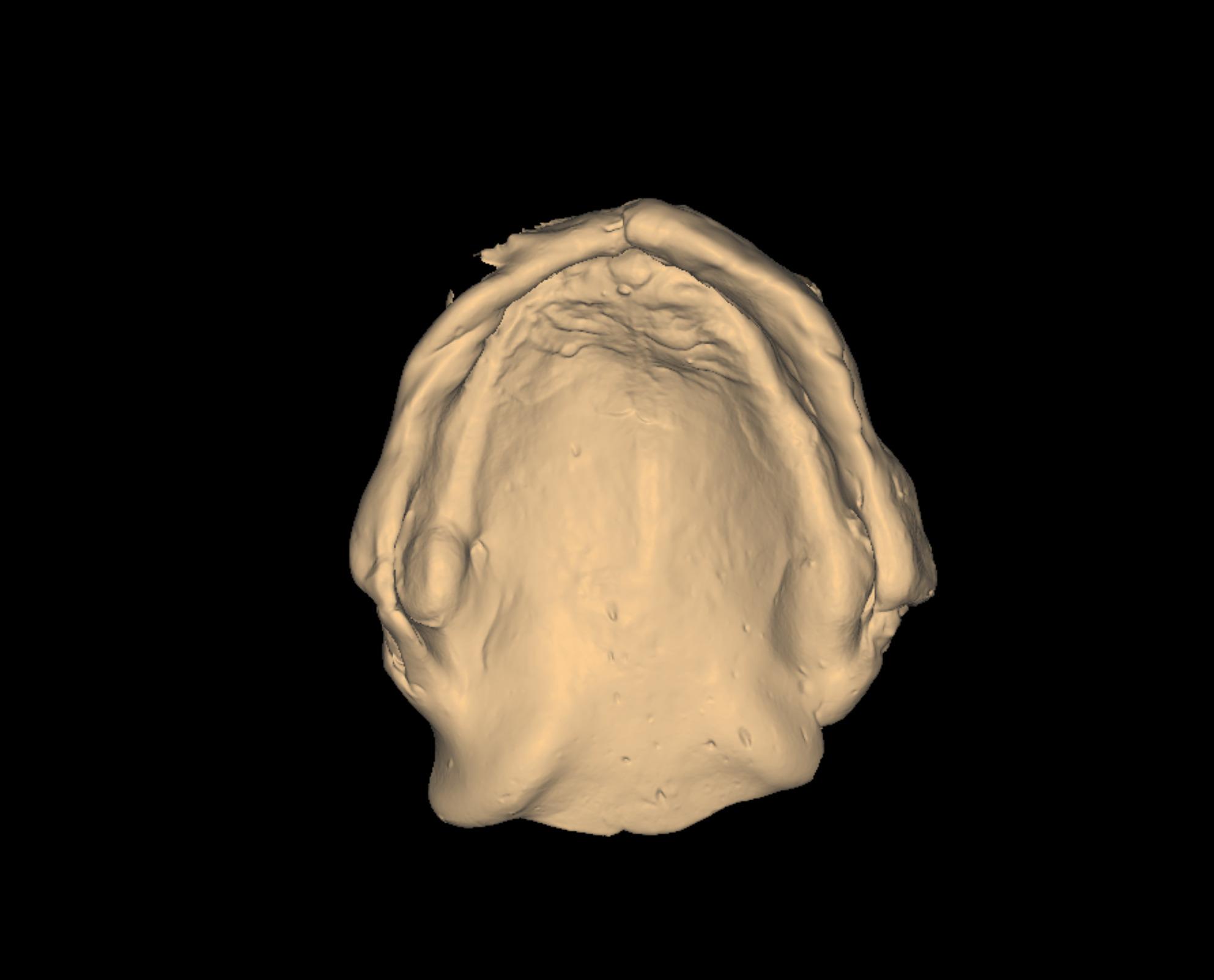
Window technique impression scan

Border molding of the custom trays were made using tracing compound (Hiflex Thermoplastic impression green sticks, Prevest Denpro) to capture functional extension. Then the custom tray was opened in the area of flabby tissues guided by the indelible pencil marks on the primary cast. Zinc oxide eugenol paste (Kelly’s Zn Oxide and Eugenol impression paste, Water Pik, Inc.) was loaded onto the tray to record the non-flabby tissues, ensuring detailed and stable impressions of these areas. A light coat of impression plaster was applied on the flabby ridge area using a small brush through the window area. Because the window exposes the flabby tissue, plaster captures its detail without compressing or displacing it, thereby preventing dislodgement of the denture during function and improving stability. (Fig. 3) The window technique impression was then scanned using a desktop extra-oral scanner (3Shape D2000 Lab Scanner, Denmark). The scanner is equipped with four 5 MP cameras and Blue LED Multi-line technology, achieving an accuracy of 5 μm (ISO)/8 μm. Prior to analysis, artifacts such as air bubbles and voids on the palatal surface of the WTI models were digitally removed to fabricate a 3D-printed model (IFUN Dental Mould resin, IFUN). (Fig. 4)

**Fig. 5:**
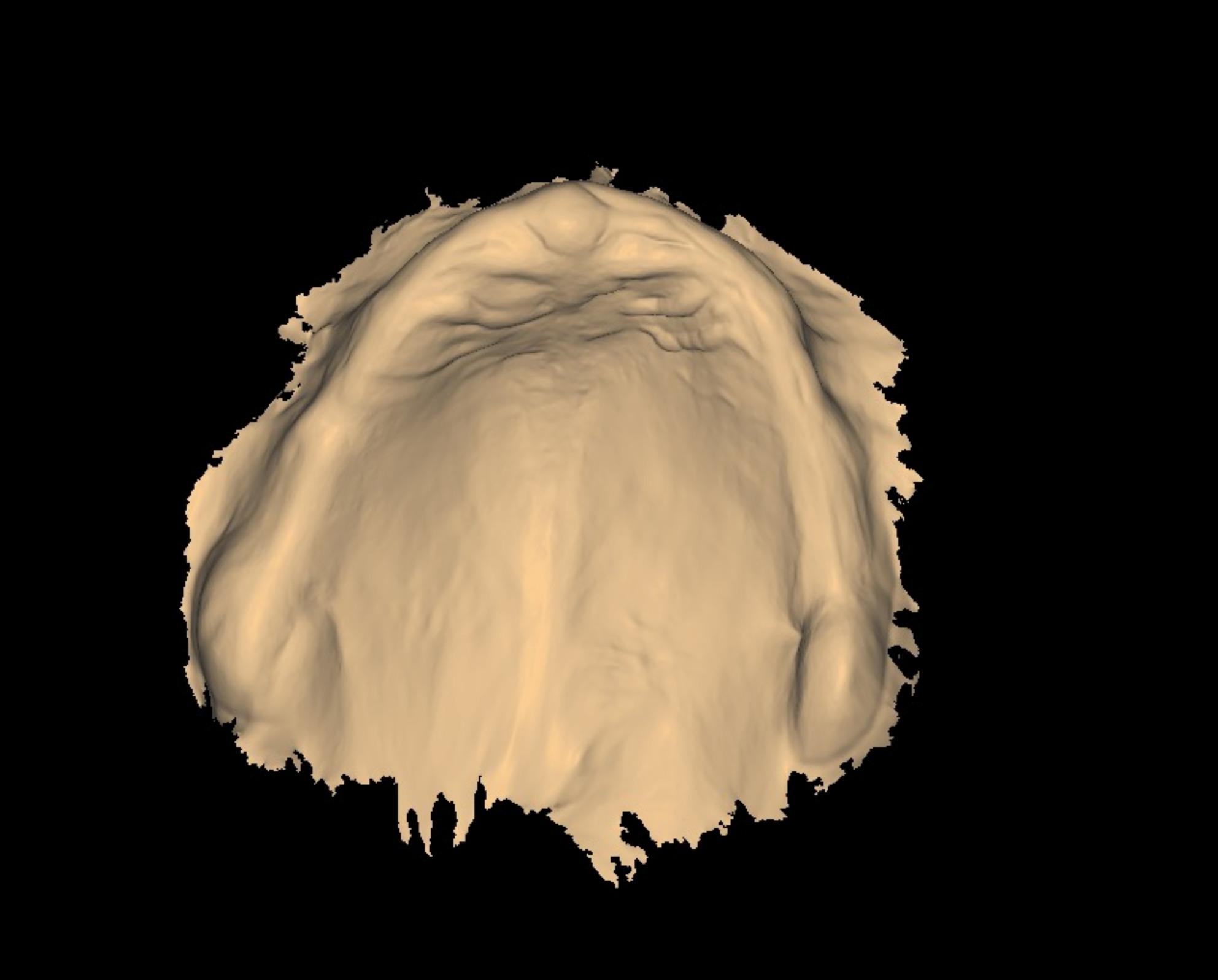
Intraoral scan

**Fig. 6:**
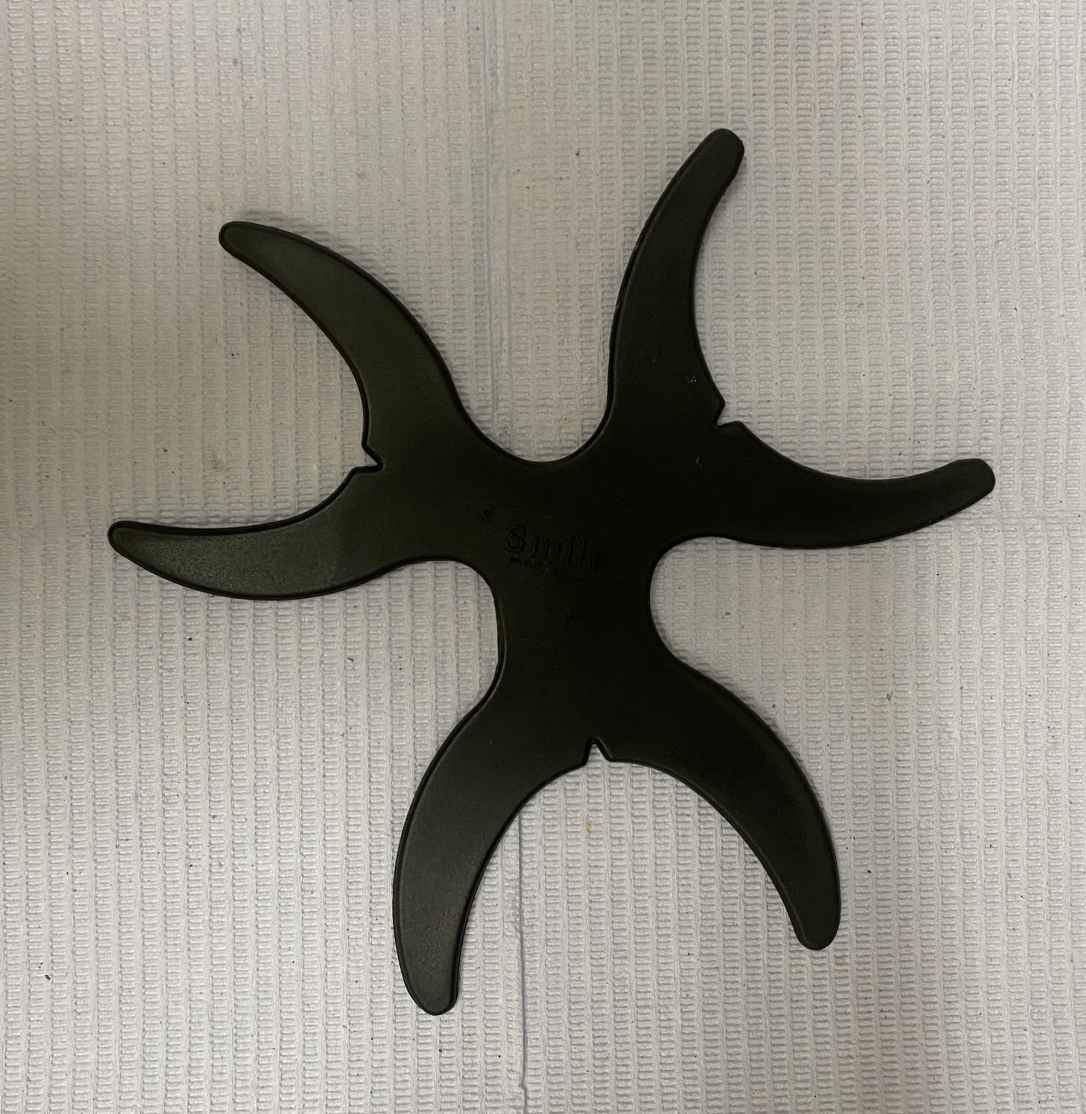
A specialized soft tissue retractor

For Group II dentures, a digital impression of maxillary arch was made using an IOS (TRIOS; 3Shape A/S) to obtain the test files (Fig. 5). A scan retractor (Flexi Triple C Retractor, Polyamide PA06, DRdent products inc, 10th of Ramadan, Egypt) was constructed with a framework adequately resilient to fit into the vestibular area, allowing unhindered movement of the scanner head which is essential for obtaining precise intraoral data without soft tissue interference [16]. (Fig. 6) Before scanning, a thorough cleansing of the edentulous ridge and removal of any residual saliva were accomplished.

**Fig. 7:**
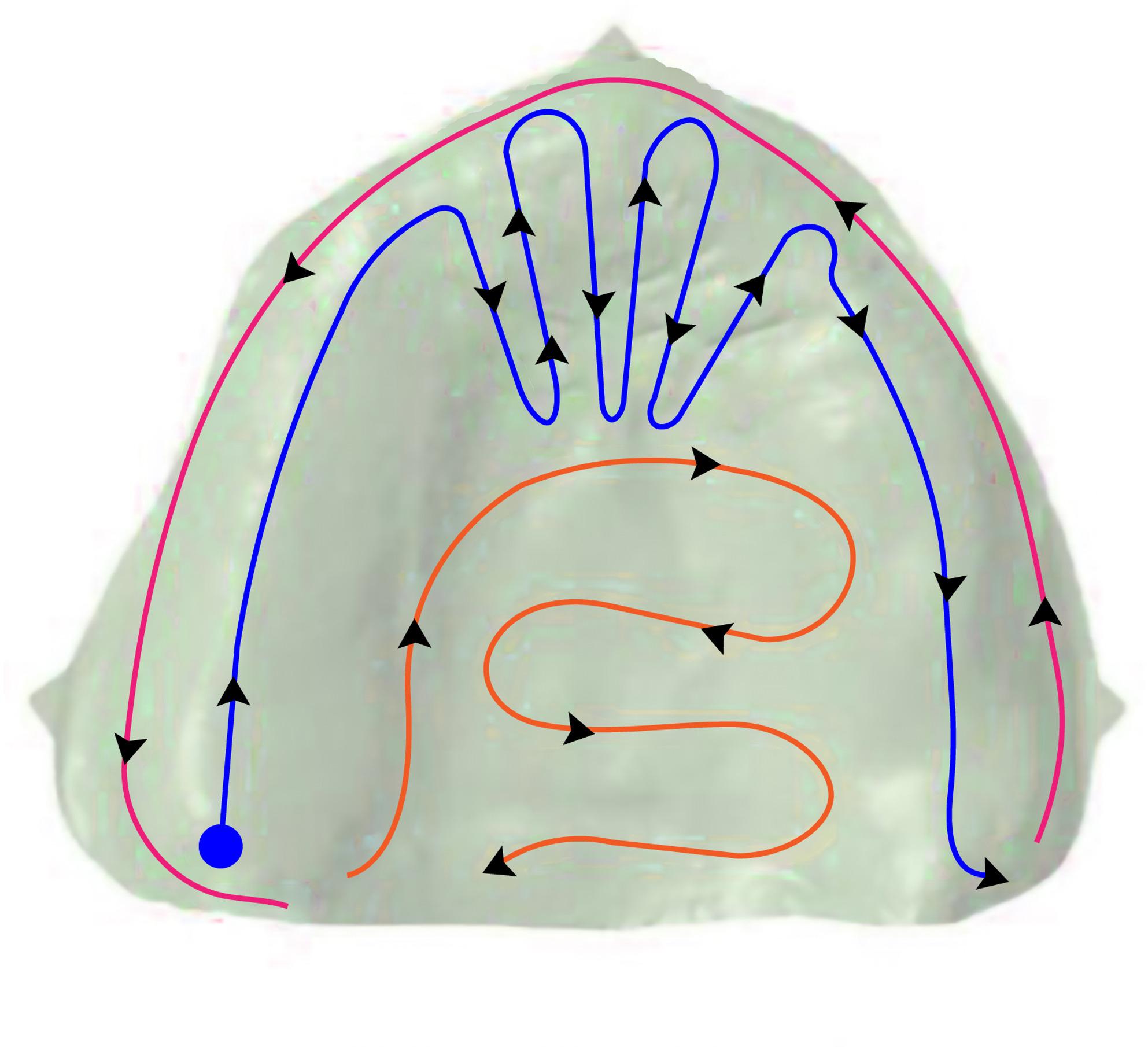
Diagram for the scanning pathway employed

**Fig. 8:**
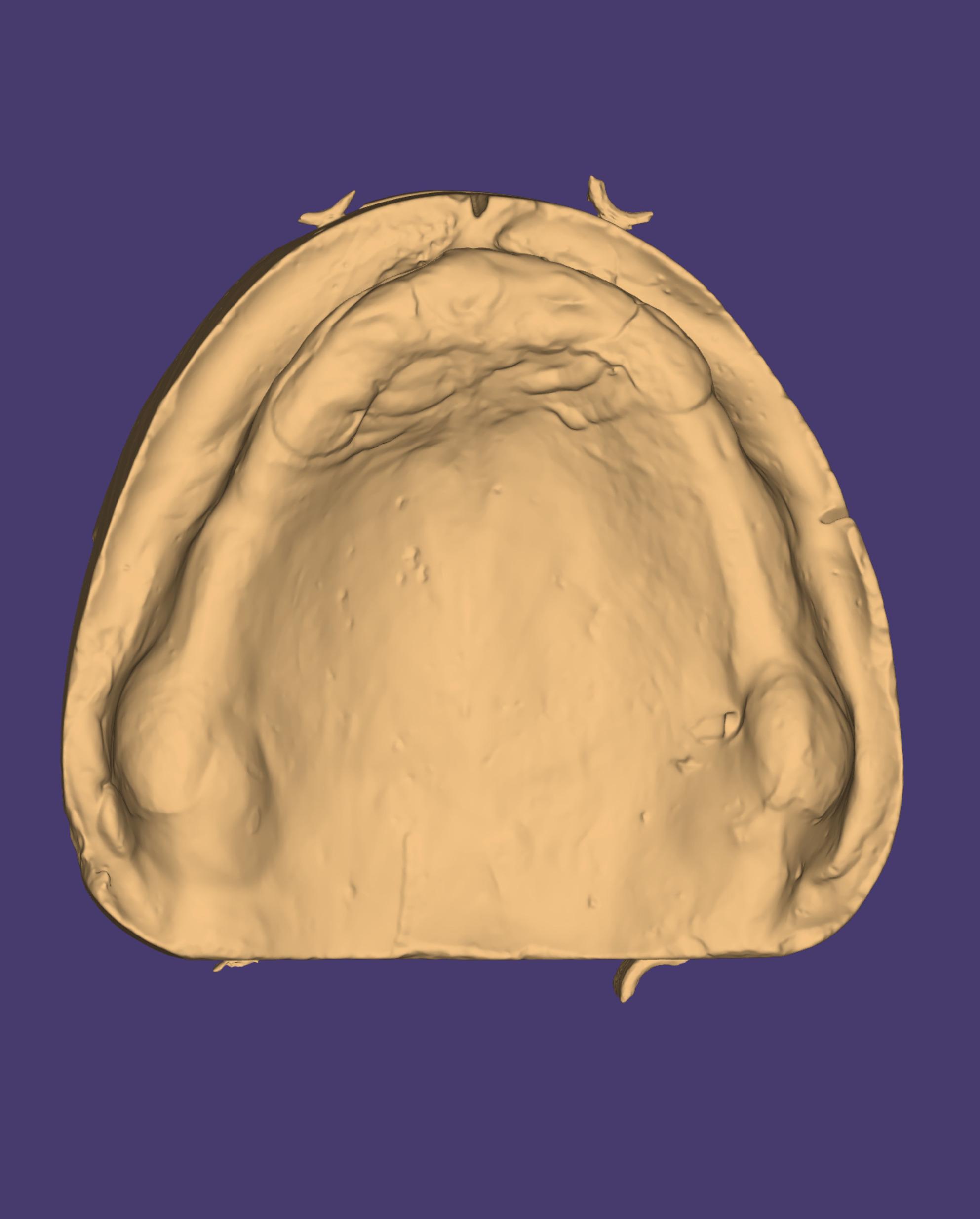
Model created on software

A systematic scanning pathway was employed for scanning the maxillary edentulous ridge. The scanning started at the ridge’s crest on one side and proceeded along the residual ridge until reaching the canine-incisor region, then a zig-zag scanning pattern was used to capture both the ridge crest and the anterior palatal slope, then the scanning continued in a straight line along the ridge’s crest to the other side. Consequently, the scanner head was turned to scan the buccal aspect, moving in a straight line to the opposite buccal side. The palatal incline of the tuberosity was then captured and the scanner was advanced anteriorly until it reached the previously scanned anterior palatal slope; then continued in a backward direction across to the opposite side, ensuring complete coverage of the entire palatal region. (Fig. 7) The systematic scanning pathway used for all cases is important for the creation of accurate digital models that form the basis of manufactured prosthesis [17]. (Fig. 8)

**Fig. 9:**
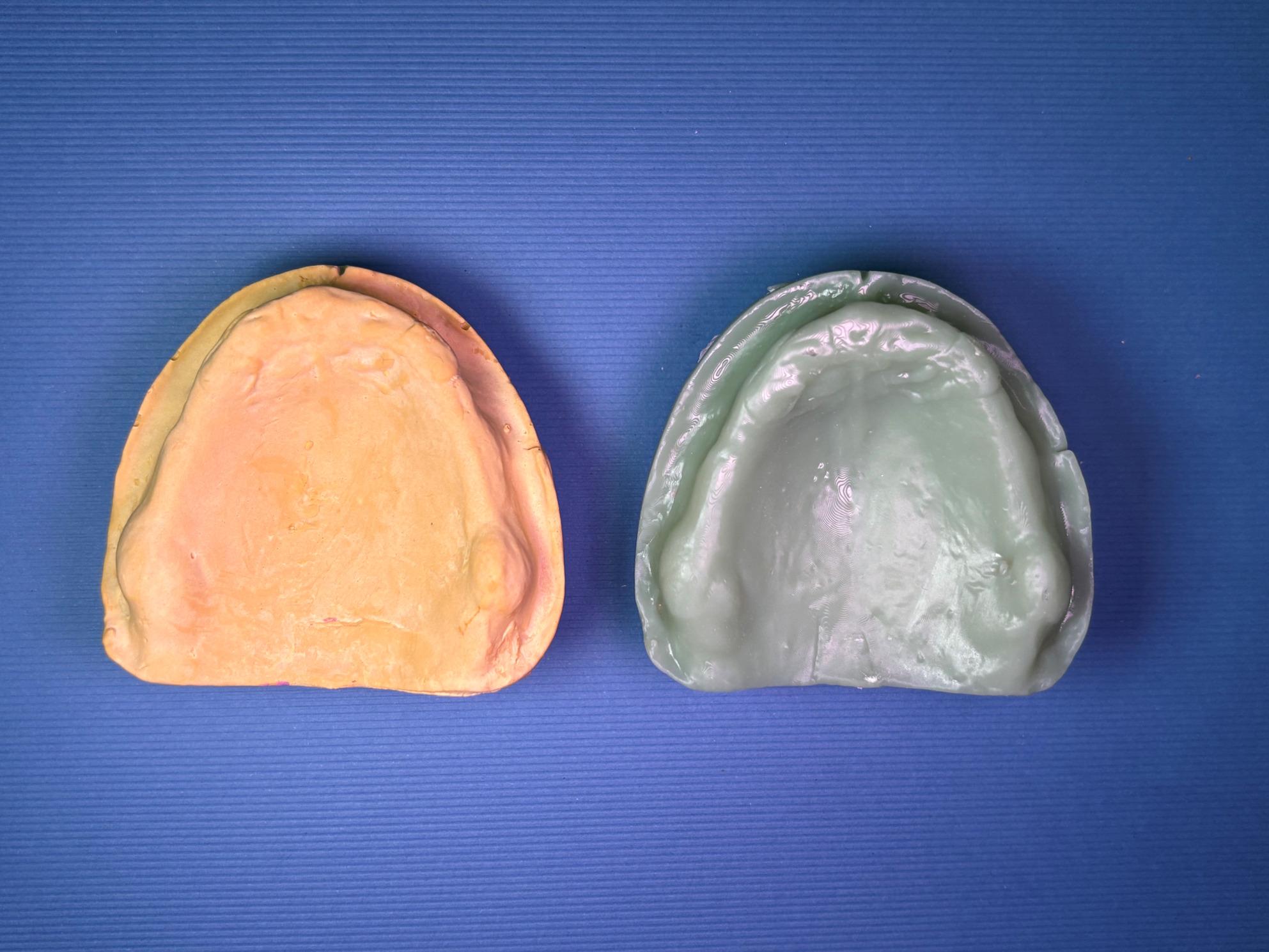
3D-printed cast duplicated into stone cast

The resultant STL files of the scan of the window technique impression and the IOS of the maxillary arch were exported to a RapidShape D30 2016 3D-printer (Rapid Shape, Heimsheim) to produce 3D-printed models of the maxillary arch. The 3D-printed models were then duplicated (Fig. 9) by enclosing the models within a mold box ensuring it is positioned securely. Silicone molding material (Heraform RS, Kulzer) was mixed and poured into the box completely encasing the 3D-models until the silicone was cured. The 3D-printed cast was carefully removed from the silicone mold. Type III dental stone was mixed and poured into the silicone mold ensuring it filled all fine details and allowed to harden completely.

For both Groups, the final impression of the mandibular arch was made using border molding with green stick compound and Zn oxide and eugenol final impression material. All the conventional steps of complete denture construction then proceeded [14]. Denture fabrication process proceeded with bite registration, where meticulous adjustment of the maxillary occlusal rim ensured proper lip support and incisal edge position. The occlusal plane level was then precisely determined using a Fox plate (Fox rule, Bio-Art A7 Plus, Italy). The Vertical Dimension of Rest (VDR) was established by measuring the distance between reference points on the nose and chin while the patient wore the maxillary rim, and the Vertical Dimension of Occlusion (VDO) was set to be 3 mm less than the VDR. The Centric Relation was recorded by manually guiding the mandible to its most retruded position. Key aesthetic landmarks, including the midline, smile lines, and canine lines, were subsequently marked on the occlusion rims. The maxillary cast was mounted onto the articulator (Bio-Art A7 Plus Articulator, Italy) using a maxillary face bow, and the mandibular cast was mounted using the centric jaw relation record. The selected cross-linked acrylic resin teeth (Vitapan®; Vita Zahnfabrik-Germany) were then arranged to achieve bilateral balanced occlusion. After the initial wax-up, the denture was verified in the patient’s mouth before being processed using the compression molding technique with conventional heat-cured acrylic resin (Heraeus Kulzer GmbH, Germany). The dentures for both groups (Conventional and Digital) were fabricated by a single, experienced dental technician. The technician was not blinded to the impression type but was instructed to use identical materials and processing techniques for the final dentures. The IOS operator (D.E.) had extensive prior experience before the trial commenced, thereby minimizing the impact of the learning curve on the results. A single operator performed all scans to reduce inter-operator variability.

On the day of denture delivery, the complete denture was checked for fit, borders, extensions and occlusion. Group I dentures were delivered to the patients. After 6 months of functional denture use and a washout period of 48 hours, Group II dentures were delivered.

### Outcomes

The primary endpoint of this crossover trial is the difference between the two techniques (Digital vs. Conventional) in OHRQoL as measured by the OHIP-EDENT-19 questionnaire at T0, T3 and T6. The secondary endpoint is the difference in maxillary denture retention at the same time points.

Each patient was asked to fill a questionnaire at T0 and T3, T6. OHIP-EDENT-19 was used to assess the OHRQoL (Supplementary 1). We used the Arabic version of OHIP-EDENT-19, whose psychometric properties in completely edentulous older adults have been validated by (Haddad et al., 2020). The Arabic version of the OHIP-EDENT-19 demonstrated excellent internal consistency (Cronbach’s α = 0.929) and high reproducibility (ICC = 0.922) in a Lebanese edentulous sample (*n* = 202). It also showed good discriminant validity relative to prosthetic variables (*p*< 0.05) [18]. (Supplementary 2). The profile consisted of 19 questions, each with a score of 0 to 4 (impaired) on the Likert scale.

The denture retention was measured at T0, T3, and T6 by a digital force gauge device (Extech’s Model 475055 Digital Force Gauge; FLIR Commercial Systems), which measures tension or compression force (pull/push) to values up to 980 N. The snap loop of the device engaged a screw that was fixed to the geometric center of the denture bases with self-curing acrylic resin to avoid heat-induced distortion of the denture base, thus maintaining the integrity of the base adaptation. The geometric center is typically located by marking the midline of the maxillary cast, drawn from the center of the incisive papilla extending posteriorly to the midpoint of a line connecting the two hamular notches. The midpoint on this midline represents the geometric center of the maxillary arch [19]. This point is used as a stable and balanced reference for placing attachments or screws.

The rigor of the outcome measurement for denture retention was ensured by following a standardized, quantifiable methodology utilizing a digital force gauge. Retention was evaluated by performing five consecutive pulls at each designated time point [20, 21]. For each pull, the peak dislodging force (the maximum resistance recorded before complete displacement) was registered by the gauge [22]. A rest interval of 60 s was enforced between each pull, allowing for soft tissue recovery and the re-establishment of the crucial salivary film required for reliable physical retention measurement [23]. The retention force was applied to a custom-fabricated attachment loop on the denture and pulled by the force gauge in a vertical direction, directly opposite to the denture’s path of insertion [22, 24]. To eliminate kinetic or inertial errors, the force was applied at a constant, slow rate of approximately 50 mm/minute until dislodgement [25]. Finally, the reported retention value for each time point was calculated as the mean (average) of the peak forces recorded from the five consecutive pulls, which is a common practice to minimize measurement variability and ensure the final reading is highly representative of the prosthetic retention [21, 25]. The device was used within its manufacturer-recommended calibration cycle to ensure accurate measurement. The instrument was newly calibrated prior to the start of the trial.

A single, calibrated examiner performed all measurements; thus eliminating inter-examiner variability. The intra-examiner reliability was assessed prior to the study using the Intra-class Correlation Coefficient (ICC), which showed high reliability (ICC > 0.90).

### Statistics

Outcomes A statistical analysis plan was performed. (Supplementary 3) Statistical analysis was performed with SPSS 27^®^, Graph Pad Prism^®^ and Microsoft Excel 2016. All data were tested for normality by Shapiro Wilk and Kolmogorov. Comparison between groups was made using paired t-test, while comparison between different time points was performed by using Repeated Measures ANOVA test followed by Tukey’s Post Hoc test. The significant level was set to be at *P* ≤ 0.05. A sensitivity analysis was performed for period 1 data to mitigate the influence of crossover design.

A Minimal Clinically Important Difference (MCID) has not yet been established for the OHIP-EDENT-19 questionnaire. Therefore, between-group differences in OHRQoL were interpreted using statistical significance and the magnitude of observed score changes, in line with recommended practice when an MCID is unavailable.

## Results

All patients completed the follow up period with no dropout. The mean age of participants was 56 ± 2.4 including 12 males and 14 females with a period of edentulism of 5 ± 2.6. Mean and standard deviation of OHIP-EDENT-19 questionnaire in both groups at different time points are presented in Table 1. The scoring system for the OHIP-EDENT-19 used a Likert scale where a lower score indicates a better perceived OHRQoL, as the minimum possible score is 0 and the maximum is 76 (higher score indicating more impairment). Normality test revealed that all data were normally distributed. Intragroup comparisons between different time points within both groups were performed by using Repeated Measures ANOVA test which revealed that there was a statistically significant improvement in OHRQoL scores over time within both groups (*P* < 0.0001). On the other hand, there were no statistically significant intergroup differences at baseline and 6 months with *P*-value 0.88 and 0.42 respectively. There was a statistically significant difference at 3 months with *P*-value = 0.001 (Table 1).

**Table 1:** Mean and standard deviation of OHIP-EDENT-19 questionnaire in both groups at different time points

OHRQOL	Group				Paired Differences						Effect size			
	Group I		Group II		Mean	Std. Deviation	Std. Error Mean	95% Confidence Interval of the Difference		*P*-value	Cohens D	Point Estimate	95% Confidence Interval	
	Mean	Standard Deviation	Mean	Standard Deviation				Lower	Upper				Lower	Upper
Baseline	57.38 ^a^	11	57.15 ^a^	9.3	0.23	7.45	1.46	−2.78	3.24	0.88	7.45	0.03	−0.35	0.42
3 months	46.27^b^	3.72	44.58^b^	3.09	1.69	2.28	0.45	0.77	2.61	0.001*	2.28	0.74	0.30	1.17
6 months	33.88 ^c^	2.63	33.81^c^	2.68	0.08	0.48	0.09	>−0.12	0.27	0.42	0.48	0.16	−0.23	0.54
*P*-value	<0.0001*		<0.0001*											
% of change	−38.13	17	−39.21	11.7	1.07	11.56	2.27	−3.60	5.74	0.64	11.56	0.09	−0.29	0.48

*Significant difference as *P* ≤ 0.05

Means with different superscript letters were significantly different as *P* <0.05

**Fig. 10:**
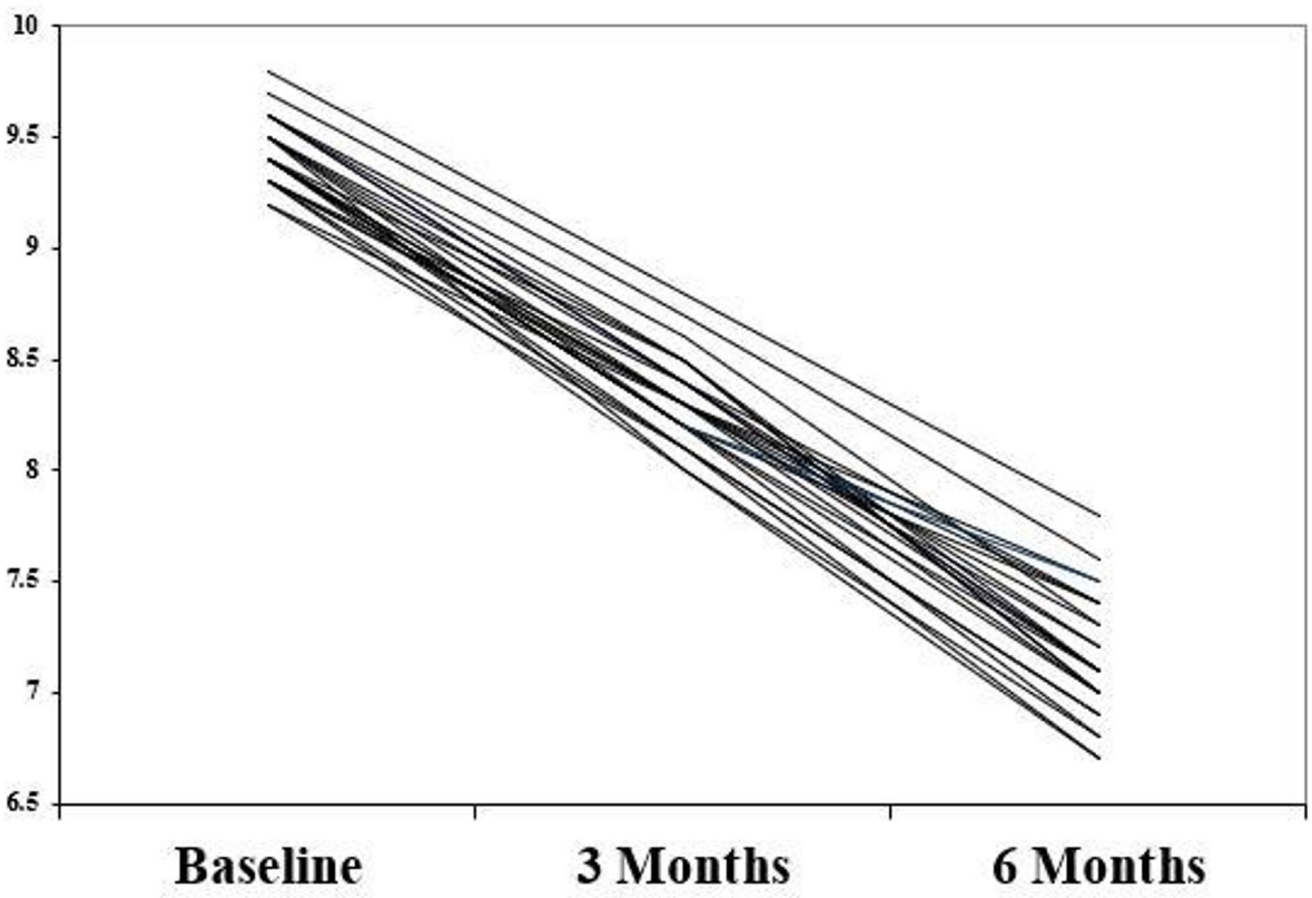
Individual retention values in the study population over the study period

**Fig. 11:**
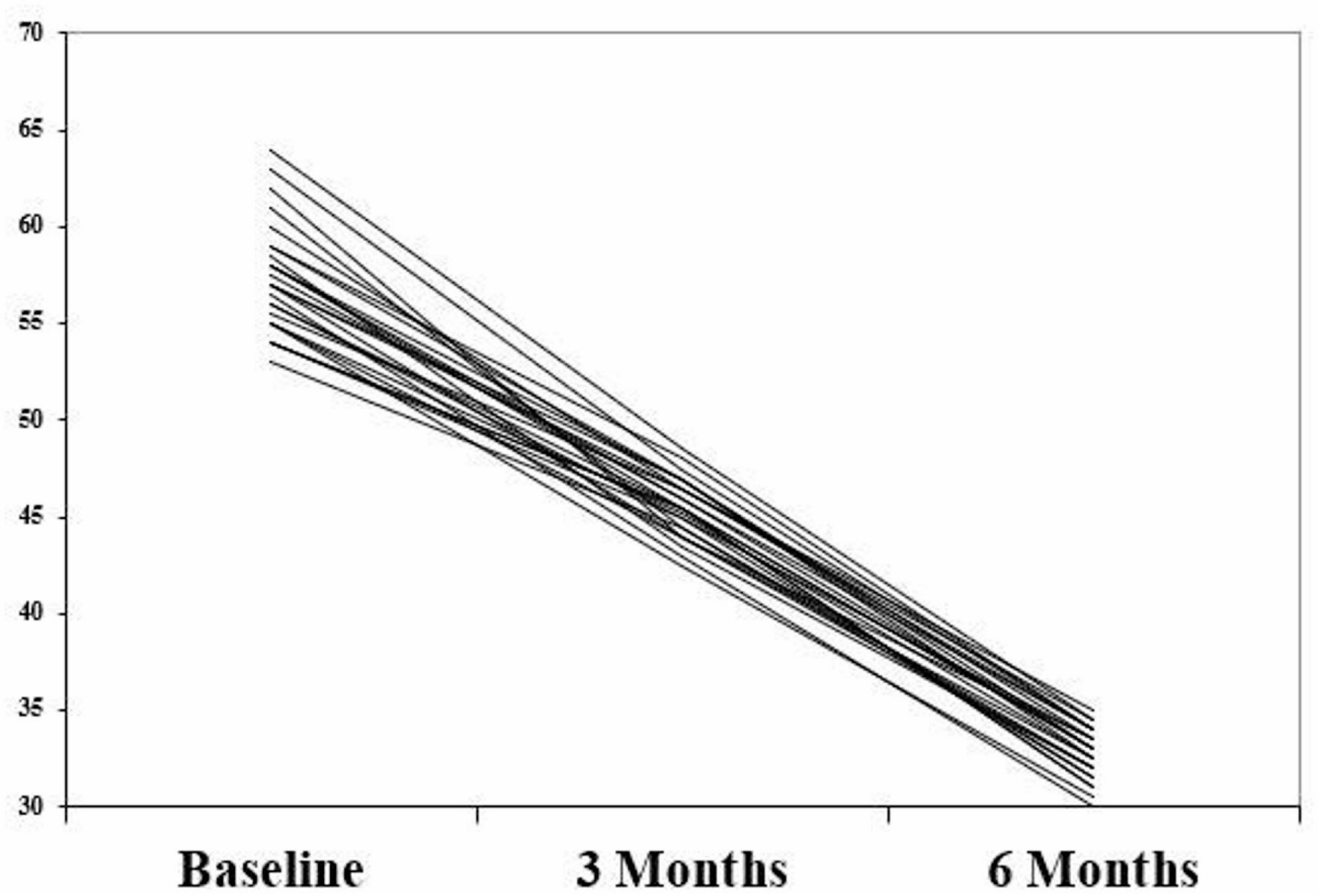
Individual QHRQOL values in the study population over the study period

 Mean and standard deviation of retention in both groups at different time points were presented in Table 2. Intragroup comparisons between different time points within both groups were performed using Repeated Measures ANOVA test which revealed that there was a significant decrease in retention over time in both groups (*P* < 0.0001). On the other hand, there were no statistically significant intergroup differences at any time points (P-value ranging between 0.08 and 0.10) (Table 2). Individual retention and QHRQOL values in the study population over the study period presented in spaghetti plots (Figs. 10 and 11). Sensitivity analysis after period 1 revealed the same results as the study design. (Table 3)

**Table 2:** Mean and standard deviation of retention (in Newton (N)) in both groups at different time points

Retention	Group				Paired Differences					*P*-value	Effect size		
	Group I		Group II		Mean	Std. Deviation	Std. Error Mean	95% Confidence Interval of the Difference		Cohens D	Point Estimate	95% Confidence Interval	
	Mean	Standard Deviation	Mean	Standard Deviation				Lower	Upper			Lower	Upper
Baseline	9.48 ^a^	0.35	9.39 ^a^	0.38	0.08	0.1	−0.12	0.29	0.4	0.22	0.04	0.00	0.17
3 months	8.38 ^b^	0.35	8.30 ^b^	0.43	0.07	0.11	−0.14	0.29	0.5	0.21	0.04	−0.01	0.16
6 months	7.00 ^c^	0.6	6.90 ^c^	0.55	0.1	0.16	−0.22	0.42	0.52	0.23	0.04	0.01	0.20
*P*-value	<0.0001*		<0.0001*										
% of change	−26.18	4.71	−26.56	4.95	0.38	1.34	−2.31	3.07	0.78	2.03	0.40	−0.44	1.20

*Significant difference as* P* ≤ 0.05

Means with different superscript letters were significantly different as *P* <0.05

**Table 3:** Sensitivity analysis using period-1 data only

		group					Paired Differences				
		Group I		Group II		Mean	Std. Deviation	Std. Error Mean	95% Confidence Interval of the Difference		*P*-value
		Mean	Standard Deviation	Mean	Standard Deviation				Lower	Upper	
Retention	baseline	9.50	0.36	9.33	0.42	0.17	0.29	0.08	−0.01	0.34	0.057
	3 months	8.40	0.36	8.25	0.51	0.15	0.29	0.08	−0.03	0.32	0.095
	6 months	7.05	0.64	6.85	0.55	0.21	0.29	0.08	0.03	0.38	0.024
	%	−25.82	4.97	−26.58	5.47	0.76	2.87	0.80	−0.98	2.49	0.359
OHRQOL	baseline	57.38	11.23	57.92	7.80	−0.54	8.62	2.39	−5.75	4.67	0.826
	3 months	46.31	3.77	44.15	3.05	2.15	2.51	0.70	0.64	3.67	0.009
	6 months	34.23	2.68	34.08	2.81	0.15	0.69	0.19	−0.26	0.57	0.436
	% of change	−37.02	20.19	−40.30	8.68	3.28	14.88	4.13	−5.71	12.27	0.442

*Significant difference as *P*≤ 0.05

## Discussion

This study aimed to compare the OHRQoL and retention of maxillary dentures fabricated from window impression technique versus IOS technique in completely edentulous patients with maxillary flabby ridges. Based on the results of the study, no evidence of a difference within the precision of this study between groups was revealed regarding the OHRQoL and retention of maxillary dentures at six months follow-up period, so the null hypothesis was accepted. Sample size calculation only ensured 90% power for a paired t-test and the study may be underpowered to detect smaller, yet clinically relevant, effects. 

The systematic review accomplished by Srivastava et al. and published in 2023 found that the TRIOS scanner was the most frequently used scanner in clinical studies evaluating edentulous arch impressions [26]. In our study based on this systematic review and another systematic review made by Wang et al. in 2024, [27] we employed the TRIOS, 3Shape A/S scanner. Given its proven frequency in related studies, its high accuracy for edentulous arches, and its evident advantages for patient comfort and clinical effectiveness, we selected the TRIOS 3Shape A/S scanner for our study as it offers reliable performance and enhance the overall clinical workflow.

To ensure uniformity in the lower arch impression technique among the two groups, the mandibular arch impression was made using the conventional method using border molding with green stick compound and zinc oxide eugenol final impression material. 3D-printing of the maxillary models allowed the production of highly accurate and precise models, by directly converting the digital and conventional impression data into a physical form with minimal dimensional errors, which eliminated this confounder when comparing between groups [28, 29]. Silicon molding material was used to duplicate the 3D-printed casts then pour them with Type III dental stone to create working casts suitable for dentures processing [30]. This indirect method ensures the preservation of the accurate details captured by the impressions. 

For an accurate assessment of the two impression methods, this clinical trial’s evaluation of OHRQoL and maxillary denture retention is important. One of the main factors influencing the clinical success of a denture is its retention, which directly impacts on the patient’s ability to speak, eat, and feeling confident [31]. Insufficient retention is thus an important measure in this study since it causes denture instability, discomfort, and functional limitations. However, the patient’s subjective experience is a crucially important factor in evaluating the success of a denture, complementing its clinical performance. OHRQoL evaluates how oral health conditions affect a person’s overall well-being, taking into account social, psychological, and physical aspects of day-to-day living [15]. By including OHRQoL as an outcome, this study acknowledges that improving a patient’s overall quality of life, rather than merely making a mechanically satisfactory denture, is the main objective of prosthetic treatment. Therefore, by combining the objective assessment of retention with the subjective evaluation of OHRQoL, a better patient-centered understanding of the clinical effectiveness of digital versus conventional impression techniques in the challenging situation of flabby ridges can be obtained.

A 48-hour washout period was adopted [13, 32] based on the distinction between biological (soft-tissue related) carryover effects and psychological or perceptual carryover effects associated with the study’s patient-reported outcomes (comfort, function, and OHRQoL) [33]. Because these outcomes are strongly influenced by patients’ short-term perceptual adjustment and immediate comparative memory of the prostheses, the associated carryover is predominantly psychological and diminishes rapidly. A 48-hour interval without wearing the first set of dentures is therefore considered sufficient to reduce perceptual bias and re-establish a neutral subjective baseline for evaluating the second device. Additionally, extending the washout period was deemed socially and ethically problematic, as prolonged edentulism can increase the risk of nutritional compromise in this population [34]. From a biological perspective, while long-term soft-tissue adaptation relevant to denture retention occurs over the six-month wear period, short-term mucosal changes induced by the preceding prosthesis are expected to resolve within 48 h [35]. Thus, the selected washout period allows the oral mucosa to return to a comparatively rested state, ensuring that the retention of the second denture reflects its fit to the existing tissue contours and providing a more standardized basis for comparison.

The study’s observation of a statistically significant decline in maxillary denture retention over the six-month period for both groups (*P* < 0.0001) warrants a deeper exploration of the underlying biological and mechanical factors. The most prominent cause of long-term retention loss in complete dentures is the irreversible process of alveolar ridge resorption, which is continuous and alters the fitting surface, gradually reducing the peripheral seal [36]. Furthermore, the presence of a flabby ridge introduces a compounding factor; the mobile, fibrous tissue is susceptible to creep and deformation under occlusal loading, which can lead to reduced efficacy of the impression surface over time, diminishing retention [37]. The constant, subtle movement of the denture base against the underlying tissue likely contributes to this progressive decline. To better understand the clinical relevance of this objective decline, we performed a correlation analysis between the change in maximum retention force and the scores of the relevant OHIP-EDENT-19 domains, specifically focusing on functional limitation [35]. 

This decline aligns with existing literature that highlights the challenges faced by completely edentulous patients, particularly those with flabby ridges, in maintaining denture stability. [5–10] Despite this decline, paired t-tests revealed no statistically significant differences in retention between the two techniques at any time point, suggesting comparable effectiveness. Factors such as the established reliability of the Window Technique in capturing anatomical nuances, minimized variability in the digital method due to a single operator performing all scans, systematic scanning pathways, and employing a specialized scanner advocated for edentulous arches may have contributed to these findings. Additionally, both groups utilized the same materials and processing techniques post-impression, which may have resulted in similar retention characteristics regardless of the initial impression method used.

In terms of OHRQoL, both groups showed significant improvements over time, with scores improving from baseline to six months (Group I: 57.38 to 33.88; Group II: 57.15 to 33.81, *P* < 0.0001). These results indicate that both impression techniques are instrumental in effectively enhancing OHRQoL, which is critical in dental care. The absence of significant differences in OHRQoL scores between the groups after the 6 months follow up period emphasizes that factors beyond the impression technique such as patient adaptation, psychological well-being, and overall treatment satisfaction play crucial roles in perceived quality of life even with decreased physical retention. This suggests that both conventional and digital methods can foster similar levels of OHRQoL, reinforcing the need for individualized treatment approaches that consider unique patient circumstances.

 While a non-statistically significant difference was revealed between groups in the OHRQOL and dentures retention; patient centered secondary endpoints revealed significant differences. Patients preferred the dentures fabricated using IOS which typically required less chairside time, less discomfort and fewer gagging incidents by eliminating the physical impressions. On the other hand, both dentures reported similar rates of adverse events, ulcerations, and dentures adjustments indicating that both dentures didn’t notably decrease adverse events. These findings suggest that while both impression techniques yield comparable clinical outcomes, the advantages of digital impressions could significantly enhance the edentulous patient experience in practices.

### Limitations

One of the study’s limitations was the potential for Type II errors, which happen when variations go unnoticed, due to an insufficient sample size. A larger sample size would allow for stronger statistical power and more reliable findings. Furthermore, the six-month evaluation period may not adequately reflect long-term changes in OHRQoL or denture retention. Significant changes in ridge morphology caused by gradual changes in soft tissue adaptation, such as the formation of flabby ridges, or progressive bone resorption may require a longer follow-up. Accordingly, longer follow-up times might provide more clinically significant data.

Because no validated Minimal Clinically Important Difference (MCID) is currently available for the OHIP-EDENT-19, direct interpretation of the clinical meaning of between-group score differences relied on effect sizes rather than predefined thresholds. In this study, the between-group differences were small in magnitude and statistically non-significant, suggesting that the absence of statistical significance also reflects a lack of clinically meaningful difference. Importantly, both impression techniques produced substantial within-group improvements in OHRQoL over time, indicating meaningful patient benefit irrespective of the method used.

 While the crossover design poses a risk of carryover effects, we implemented a robust sensitivity analysis to assess and mitigate this risk, ensuring the reliability of our findings. Additionally, we actively monitored blinding throughout the trial; although the indistinguishability of dentures helped maintain blinding, we recognize that factors such as participant biases could still arise in subjective assessment. Moreover, to combat potential operator dependence, we implemented rigorous training protocols and standardized scanning pathways for the IOS operator, minimizing variabilities and enhancing consistency in measurements. Finally, we are committed to transparency concerning the retrospective registration of our study; to mitigate potential reporting biases, we have thoroughly adhered to and reported all outcomes as established in our pre-specified protocol.

### Recommendations

- Regular monitoring of denture retention is recommended to ensure patient comfort and functionality as highlighted by the decline of denture retention by time in this study.
- The improvement of OHRQoL highlights the importance of considering improving a patient’s overall quality of life and adopting a holistic approach in prosthetic treatment.
- The viability of scanning maxillary edentulous arches as reported by this study advocates further research to validate fully digital workflows, which could improve accuracy in capturing oral anatomy and streamline the denture fabrication process, ultimately benefiting patient outcomes.

## Conclusion

This randomized controlled trial reported comparable OHRQoL and retention of dentures fabricated from digital and conventional impression techniques in edentulous patients with maxillary flabby ridges. The digital impression is a viable alternative to conventional impression in the fabrication of complete dentures for edentulous arches with flabby ridges.

## Supplementary Information

Supplementary Material 1.

Supplementary Material 2.

Supplementary Material 3.

## Data Availability

All data generated or analysed during this study are included in this published article.
