# Supplementary material for: Clinical evaluation of digital versus conventional impression in edentulous patients with flabby ridges: a randomized controlled clinical trial
Source: BMC Oral Health. 2026 Jan 12;26:187. doi: 10.1186/s12903-025-07524-8 (PMC12853624; doi:10.1186/s12903-025-07524-8)
Supplement: Supplementary file 1 — Supplementary Material 1. [file 12903_2025_7524_MOESM1_ESM.pdf]

OHIP-EDENT-19 questionnaire form:

A-functional limitation.

1.Have you had difficulty chewing any food because of problems with your denture?

2. Have you had food catching in your dentures?

3.Have you felt that your dentures haven't been fitting properly?

B-physical pain.

4.Have you had painful aching in your mouth?

5.Have you found it uncomfortable to eat any food because of problems with your dentures?

6.Have you had sore spots in your mouth?

7.Have you had uncomfortable dentures?

C-Psychological disorder.

8.Have you been worried by dental problems?

9.Have you been self-conscious because of your dentures?

D- Physical disability.

10.Have you had to avoid eating some food because of problems with your dentures?

11.Have you been unable to eat with your dentures because of problems with them?

12. Have you had to interrupt meals because of problems with your dentures?

E- Psychological disability.

13. Have you been upset because of problems with your dentures?

14. Have you been a bit embarrassed because of problems with your dentures?

F- Social disability.

15.Have you avoided going out because of problems with your dentures?

16.Have you been less tolerant of your spouse or family because of problems with your dentures?

17.Have you been a bit irritable with other people because of problems with your teeth, mouth, or denture?

G-Handicap.

18.Have you been unable to enjoy other people's company as much because of problems with your dentures?

19.have you felt that life in general was less satisfying because of problems with your dentures?

A

نموذج استبيان (OHIP-EDENT-19)

أ- التقييم الوظيفي.

1-هل واجهت صعوبة في مضغ أيٍ طعام بسبب مشاكل في طقم أسنانك؟

2-هل كان لديك طعام يصطاد في أطقم الأسنان الخاصة بك؟

3-هل شعرت أن أطقم الأسنان الخاصة بك لم تكن مناسبة بشكل صحيح؟

ب- الألم القمية.

4-هل عانيت من ألم مؤلم في فمك؟

5-هل وجدت أنه من غير المريح تناول أيٍ طعام بسبب مشاكل في أطقم الأسنان الخاصة بك؟

6-هل عانيت من بقع مؤلمة في فمك؟

7-هل كان لديك أطقم أسنان غير مريحة؟

ت- التقييم النفسي.

8-هل كنت قلقا من مشاكل الأسنان؟

9-هل كنت خجولا بسبب أطقم الأسنان الخاصة بك؟

ث- المشاكل الجسدية.

10-هل اضطررت إلى تجنب تناول بعض الطعام بسبب مشاكل في أطقم الأسنان الخاصة بك؟

11-هل لم تتمكن من تناول الطعام مع أطقم الأسنان الخاصة بك بسبب مشاكل معها؟

12-هل اضطررت إلى مقاطعة وجبات الطعام بسبب مشاكل في أطقم الأسنان الخاصة بك؟

ج- المشاكل النفسية.

13-هل كنت منزعجا بسبب مشاكل في أطقم الأسنان الخاصة بك؟

14-هل شعرت بالحرج قليلا بسبب مشاكل في أطقم الأسنان الخاصة بك؟

ح- المشاكل الاجتماعية.

15-هل تجنبت الخروج بسبب مشاكل في أطقم الأسنان الخاصة بك؟

16-هل كنت أقل تسامحا مع زوجتك أو عائلتك بسبب مشاكل في أطقم الأسنان الخاصة بك؟

17-هل كنت عصبيا بعض الشيء مع الآخرين بسبب مشاكل في أسنانك أو فمك أو طقم أسنانك؟

خ- الاحتياجات الخاصة.

18-هل لم تتمكن من الاستمتاع بصحبة الآخرين بنفس القدر بسبب مشاكل في أطقم الأسنان الخاصة بك؟

19-هل شعرت أن الحياه بشكل عام كانت أقل إرضاء بسبب مشاكل في أطقم الأسنان الخاصة بك؟

B

Content courtesy of Springer Nature, terms of use apply. Rights reserved.
