# Supplementary material for: Clinical evaluation of digital versus conventional impression in edentulous patients with flabby ridges: a randomized controlled clinical trial
Source: BMC Oral Health. 2026 Jan 12;26:187. doi: 10.1186/s12903-025-07524-8 (PMC12853624; doi:10.1186/s12903-025-07524-8)
Supplement: Supplementary file 2 — Supplementary Material 2. [file 12903_2025_7524_MOESM2_ESM.pdf]

The Lebanese Arabic version of the Oral Health Impact Profile for Edentulous subjects (OHIP-EDENT-19) demonstrated good psychometric properties in a sample of 202 completely edentulous Lebanese participants. The questionnaire is comprised of 19 items grouped into seven dimensions (functional limitation, pain, psychological discomfort, physical incapacity, psychological incapacity, social incapacity, and handicap).

**Property**	**Statistical Result**	**Conclusion**
**Reproducibility** (Test-Retest Reliability)	Intra-class correlation coefficient (CCI) was **0.922**.	**Good reproducibility**.
**Internal Consistency**<br>(Cronbach's ∞)	Cronbach's Alpha coefficient (∞) was **0.929**.	**Good internal consistency**.
**Concurrent Validity**	The OHIP-EDENT score was **significantly associated** ($p<0.001$) with poor self-perceived oral health, low satisfaction with the prosthesis, perception of aesthetic problems, and the need for new prosthetic treatment.	**Good concurrent validity**.
**Discriminant Validity**	The score was **significantly associated** with prosthetic status ({p}=0.005), stability of the prostheses ($p<0.001$), number of years prostheses were worn ($p=0.004$), sex ($p=0.049$), and education level ($p=0.041$).	The questionnaire can distinguish groups based on clinical and demographic factors.
