# Supplementary material for: Clinical evaluation of digital versus conventional impression in edentulous patients with flabby ridges: a randomized controlled clinical trial
Source: BMC Oral Health. 2026 Jan 12;26:187. doi: 10.1186/s12903-025-07524-8 (PMC12853624; doi:10.1186/s12903-025-07524-8)
Supplement: Supplementary file 3 — Supplementary Material 3. [file 12903_2025_7524_MOESM3_ESM.pdf]

Supplementary 3

1. Statistical Analysis Plan

1.1 Overview

Statistical analyses were performed using SPSS 27®, GraphPad Prism®, and Microsoft Excel 2016. The analyses aimed to evaluate the primary and secondary outcomes defined in the methodology.

1.2 Normality Testing

All data were assessed for normality using the following tests:

-Shapiro-Wilk Test

-Kolmogorov-Smirnov Test

1.3 Comparison Methods

-Between Groups:Paired t-tests were utilized for comparisons between groups at the same time points.

-Across Time Points:

-Repeated Measures ANOVA followed by Tukey's Post Hoc test was employed to analyze differences across multiple time intervals.

1.4 Statistical Significance

The significance level was established at $P \leq 0.05$for all analyses.

1.5 Sensitivity Analysis

A sensitivity analysis was conducted on data from period 1 to address potential carryover effects. This included careful consideration of period, sequence, and carryover terms.

2. Primary and Secondary Outcomes

2.1 Primary Outcome

-OHRQoL Assessment:

-Differences in OHRQoL as measured by the OHIP-EDENT-19 questionnaire between Digital and Conventional techniques at T0, T3, and T6.

2.2 Secondary Outcome

-Retention Measurement:Differences in maxillary denture retention at T0, T3, and T6.

3. OHIP-EDENT-19 Questionnaire Details

3.2 Questionnaire Description

The OHIP-EDENT-19 is composed of 19 questions, scored on a Likert scale (0-4), with higher scores indicating greater impairment in OHRQoL.

-Reproducibility:ICC = 0.922 (validated in a Lebanese edentulous sample of n=202.(

-Discriminant Validity:Statistically significant relative to prosthetic variables ($p < 0.0$)

4. Denture Retention Measurement

4.1 Measurement Method

Retention was quantified using a digital force gauge (Extech's Model 475055), which measures tension or compression forces up to 980 N .

5. Data Presentation

5.1 Tables

Table 1: Mean and Standard Deviation of OHIP-EDENT-19 Scores at Different Time Points.

Table 2: Mean and Standard Deviation of Retention Measurements at Different Time Points.

Spaghetti plots illustrating individual retention and OHRQoL values over the study period.

6. Protocol Adherence

We have adhered to our pre-specified protocol to ensure transparency in reporting all outcomes. Following the recommendations, we have accurately depicted statistical methodologies and findings to minimize potential
